# Circles of deception: the Ebbinghaus illusion from fish to birds

**DOI:** 10.3389/fpsyg.2025.1653695

**Published:** 2025-10-20

**Authors:** Maria Santacà, Cliodhna Quigley, Leonida Fusani

**Affiliations:** ^1^Department of Behavioural and Cognitive Biology, University of Vienna, Vienna, Austria; ^2^Department of Interdisciplinary Life Sciences, Konrad Lorenz Institute of Ethology, University of Veterinary Medicine Vienna, Vienna, Austria

**Keywords:** visual illusion, Ebbinghaus illusion, comparative cognition, *Poecilia reticulata*, *Streptopelia risoria*

## Abstract

Understanding how animals perceive visual illusions provides valuable insights into the evolution of sensory systems and how these systems are adapted to meet the perceptual demands of an animal’s natural environment. This study investigates the susceptibility to the Ebbinghaus illusion in guppies (*Poecilia reticulata*) and ring doves (*Streptopelia risoria*), two species with contrasting ecological and sensory adaptations. The Ebbinghaus illusion, where the perceived size of a central circle is influenced by surrounding circles, offers a robust framework for exploring context-dependent size perception. Guppies displayed high susceptibility to the illusion, possibly reflecting their reliance on global visual processing for interpreting complex aquatic environments. This heightened sensitivity may reflect an adaptive response to dynamic light conditions and dense vegetation, where relative size cues facilitate tasks such as mate selection and foraging. Conversely, no consistent susceptibility to the illusion was found in ring doves, which may be attributed to their ecological adaptation as granivores, favoring local processing over global contextual integration. Such local-focused processing likely supports their need to visually discriminate discrete seeds within heterogeneous ground textures. However, high interindividual variability emerged in their responses, suggesting that factors such as past experiences or individual perceptual biases may play a role in shaping their perceptual strategies. These findings underscore the role of ecological pressures in shaping perceptual mechanisms and suggest how contrasting environmental demands can lead to diverse visual strategies even for the same illusion.

## Introduction

Understanding how animals perceive and interpret their environments is fundamental to the study of sensory ecology and evolution. Far from being a passive reception of sensory input, perception is a constructive process shaped by the interaction of sensory modalities, neural mechanisms, and ecological pressures (e.g., Feng et al., 2017; Gregory, 1997). The ability of animals to extract meaningful information from their surroundings is critical for survival and reproduction, influencing behaviors such as foraging, predator avoidance, and social interactions. One of the most intriguing ways to study perceptual mechanisms is through the investigation of visual illusions, where the perceived properties of a stimulus deviate from its physical characteristics. Indeed, visual illusions provide insights into the trade-offs between perceptual accuracy and efficiency. While distortions may seem disadvantageous, they often reflect the brain’s reliance on heuristic processing—strategies that prioritize speed and resource efficiency that sometimes sacrifice precision in favor of functionality (Gregory, 1997). These heuristics are shaped by the evolutionary and ecological pressures unique to each species, making the study of illusions a valuable approach for understanding the adaptive flexibility of perception (e.g., Feng et al., 2017; Gregory, 1997). Comparative studies of illusions highlight how perceptual systems have evolved to meet the demands of different environments, revealing conserved and divergent strategies across taxa (e.g., Agrillo et al., 2020; Feng et al., 2017).

**Figure 1:**
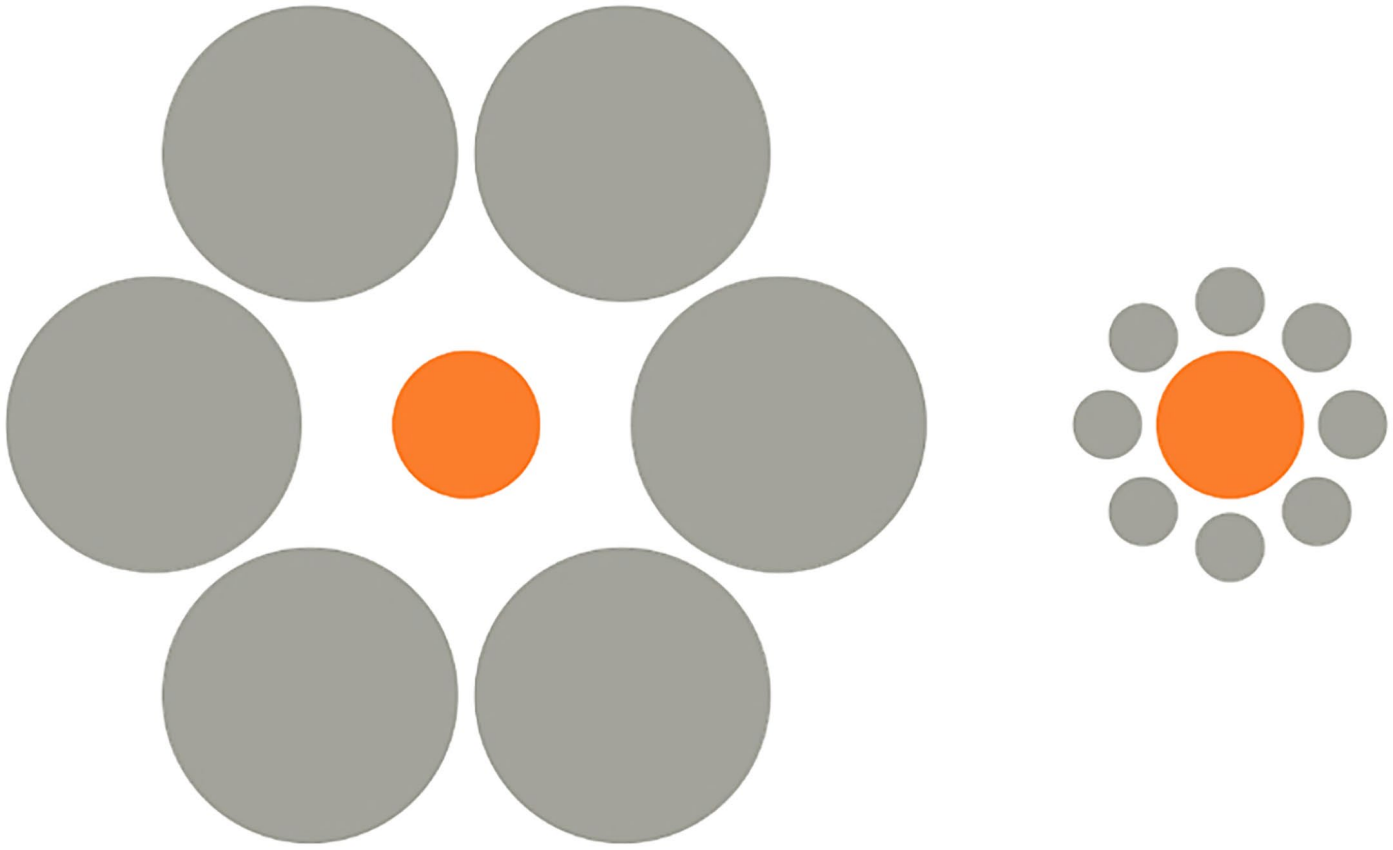
In the Ebbinghaus illusion, humans perceive as smaller a central circle when surrounded by larger inducers and, instead, they perceive it as larger when it is surrounded by smaller inducers.

The Ebbinghaus illusion is a classic example of context-dependent size perception. In humans, the perceived size of a central circle is influenced by the size of surrounding circles: the central circle appears smaller when encircled by larger circles and larger when surrounded by smaller ones (Figure 1). The perception of the Ebbinghaus illusion in humans is rooted in two key mechanisms that interact within the visual processing system: size contrast and global contextual processing. Size contrast explains the illusion as a result of the perceptual interplay between the central target and the surrounding circles, or “inducers.” When the central circle is encircled by smaller inducers, it appears larger due to the contrast effect, whereas being surrounded by larger inducers makes it appear smaller. This phenomenon is often interpreted as reflecting the general tendency of perception to rely on relative rather than absolute differences (Akre and Johnsen, 2014). However, although many findings in psychophysics support the reliance on relative differences, some studies including a recent meta-analysis by Worsley et al. (2025), show that species can differ in whether they perceive differences in a relative or absolute manner. Therefore, it is important to recognize that perceptual mechanisms may vary across species rather than being universally consistent. Global contextual processing also influences the illusion, as it involves the integration of the entire visual scene into a cohesive interpretation. Humans exhibit a strong tendency for “global precedence,” whereby the overall spatial arrangement of stimuli is processed before the details of individual elements. However, susceptibility to the illusion varies across individuals and cultures, suggesting that both innate and experiential factors shape its perception (e.g., Bremner et al., 2016, Doherty et al., 2010; see Discussion for further details).

The illusion’s reliance on contextual cues makes it a valuable tool for exploring perceptual grouping and the integration of visual information across different animal species. Comparative research in non-human animals has yielded mixed results, reflecting the diversity of perceptual strategies shaped by ecological and evolutionary pressures. For example, species such as dolphins (*Tursiops truncatus*; Murayama et al., 2012), domestic chicks (*Gallus gallus domesticus*; Rosa Salva et al., 2013), and redtail splitfins (*Xenotoca eiseni*; Sovrano et al., 2015) exhibit human-like susceptibility to the illusion. Conversely, pigeons (*Columba livia*; Nakamura et al., 2008), baboons (*Papio papio*; Parron and Fagot, 2007), and gray bamboo sharks (*Chiloscyllium griseum*; Fuss and Schluessel, 2017) show reversed effects or no susceptibility at all. These interspecific differences highlight the importance of studying visual illusions as tools for understanding not only perceptual mechanisms but also their ecological and functional significance (Kelley and Kelley, 2014).

Context-dependent size perception is not only a fascinating perceptual phenomenon but also a critical ecological skill with direct implications for survival and reproduction. For instance, in predation scenarios, accurately assessing the size of a predator relative to its surroundings can determine whether an animal chooses to flee or hide, as larger predators are perceived as a greater threat, prompting earlier escape responses, while smaller or more distant predators may elicit less urgent reactions (Cooper and Stankowich, 2010). Furthermore, in social species, size perception significantly influences dominance hierarchies, with larger individuals often perceived as more dominant. This phenomenon is well-documented in both fish and birds. For instance, in many fish species, individuals with larger body sizes tend to dominate social hierarchies, impacting access to resources and mating opportunities (e.g., Brännäs et al., 2001; Ward et al., 2006). Similarly, among birds, interspecific hierarchies are primarily organized based on body size. Research has demonstrated that body size is a significant predictor of dominance rank in avian species, influencing feeding order and access to resources and thereby affecting their social interactions and survival strategies (e.g., Moreno-Opo et al., 2020; Rabinowicz et al., 2020). These findings underscore the critical role of size perception in establishing and maintaining social structures across different taxa. Recognizing larger individuals as more dominant appears to be a common strategy that facilitates the organization of social hierarchies, influencing behaviors related to resource allocation, mating, and survival.

Beyond these specific examples, global processing—the ability to interpret an entire scene before focusing on individual elements—may offer a survival advantage in cluttered habitats where objects rarely appear in isolation, enabling animals to extract meaningful patterns from complex environments. Conversely, species that prioritize local visual features over global scene integration may show reduced susceptibility to size illusions, reflecting adaptations to tasks requiring precise discrimination of individual elements, such as locating seeds or small prey (Nakamura et al., 2008, 2014). Together, these studies emphasize the importance of investigating visual illusions as they can reveal important aspects of an animal’s ecological needs and behavioral strategies, thereby providing insight into how perceptual mechanisms have adapted to specific ecological contexts.

The present study extends this cross-species investigation to two species with contrasting ecological and sensory adaptations: guppies (*Poecilia reticulata*) and ring doves (*Streptopelia risoria*). Guppies are small freshwater fish inhabiting environments such as shallow rainforest streams characterized by dense vegetation and complex visual backgrounds (Endler et al., 2022). Their ecological niche requires precise relative size discrimination for tasks such as mate choice—where females prefer larger males—and competition, where relative size can influence outcomes. Previous research has demonstrated that guppies perceive other size illusions (reviewed in Agrillo et al., 2020 and Santacà et al., 2021) also in directions opposite to those observed in humans, suggesting the involvement of perceptual mechanisms that may be shaped by species-specific ecological constraints. By studying guppies in the context of the Ebbinghaus illusion, this research offers preliminary insight into whether their visual system integrates contextual cues similarly to other species or diverges possibly due to their aquatic habitat and specific ecological pressures. Conversely, ring doves are terrestrial birds with a foraging strategy reliant on precision and binocular vision (Baskett, 1993). Binocular vision provides stereoscopic depth perception, allowing these birds to accurately judge the distance of seeds and thus make precise absolute size evaluations, an important skill given their granivorous diet and the heterogeneous substrates they forage upon. Their visual system, while different from that of fish, is also highly adapted to their specific ecological needs. Guppies inhabit shallow streams with complex visual backgrounds and have a varied diet that includes insects, larvae, algae, and organic detritus (Magurran, 2005). Importantly, guppies forage not only on the substrate but also within the water column, feeding on particles and preys suspended in the water. This exposes them to multiple types of visual contexts, ranging from heterogeneous backgrounds on the streambed to more uniform but dynamic mid-water environments. Although no generalizations can be drawn from the comparison of just two species, birds such as ring doves offer an ecologically contrasting model to guppies. This exploratory comparison allows us to investigate whether these species process visual context differently and may help generate hypotheses about how different visual environments influence susceptibility to size illusions.

## Methods

### Ethics statement

The experiments were carried out at the Animal Care UBB Core Facility of the Faculty of Life Sciences at the University of Vienna (Vienna, Austria). This work was approved by the local ethics committee of the Faculty of Life Sciences, University of Vienna, (protocol nr. 2023–27) and adheres to the ASAB/ABS guidelines for the Use of Animals in Research, the ARRIVE guidelines and EU Directive 2010/63/EU guidelines. All efforts were made to limit any stress before and during the experiment. We monitored animals during the experiment for signs of severe stress, which could include, for doves, picking feathers, stereotypical behavior, or hissing (Miller and Miller, 1958), and, for guppies, freezings and thigmotaxis (i.e., tendency to stay close to tank walls; Houslay et al., 2022) which was never observed.

#### Subjects and experimental apparatus

Experiments were conducted on a sample of 39 adult ring doves (15 males and 24 females) and 23 guppies (6 males and 17 females), but some subjects ceased to participate in the experiment and had to be discharged. Therefore, the final sample size consisted of 38 ring doves (14 males and 24 females) and 19 guppies (2 males and 17 females). The number of individuals tested for each species was determined by availability in the research facility. Nonetheless, sample sizes were comparable or larger than those reported in similar studies on visual illusions in non-human animals (e.g., Santacà et al., 2021).

**Figure 2:**
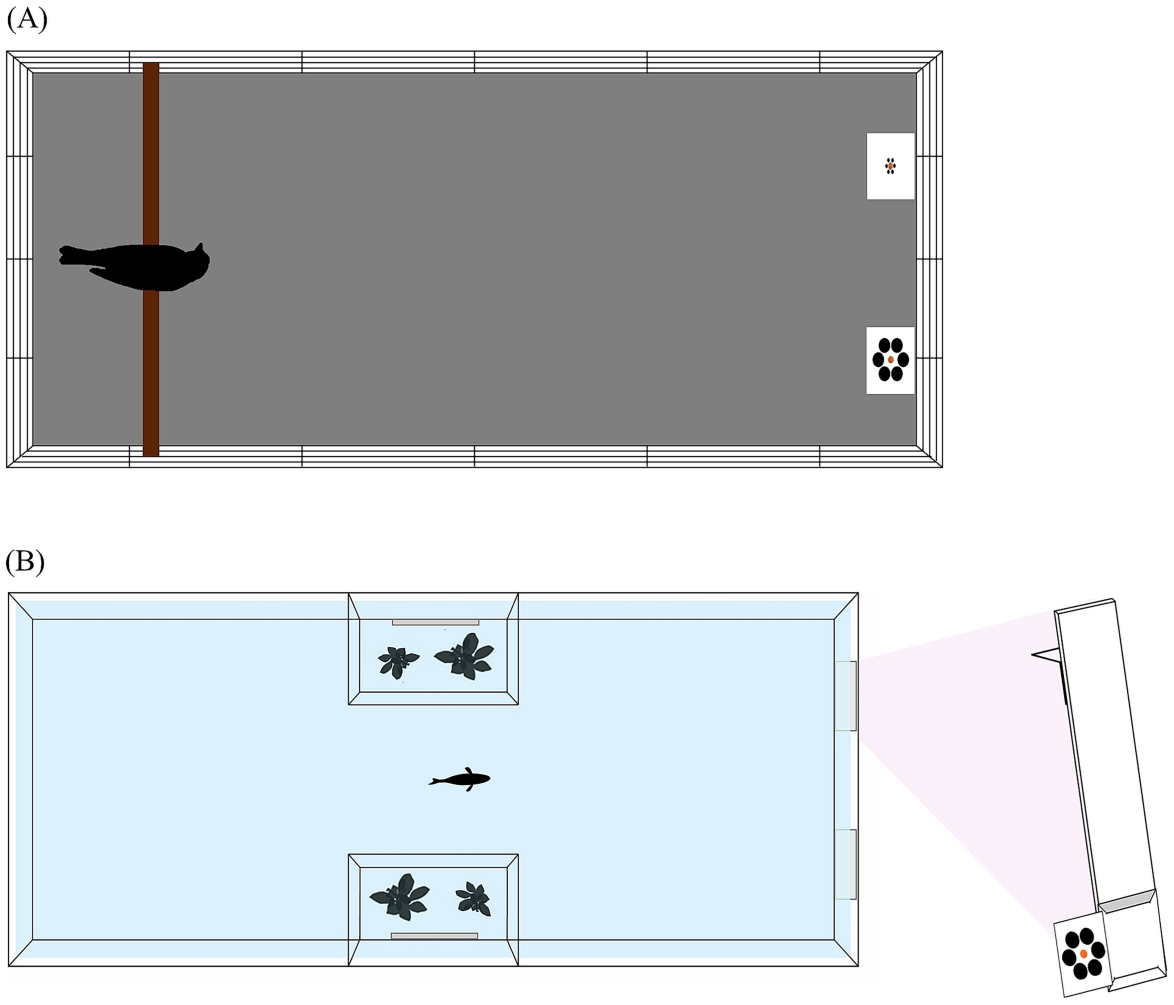
View from above of the apparatuses used for ring doves **(A)** and guppies **(B)**.

Ring doves were housed in single-sex groups in outdoor aviaries of ca. 40 m^2^ each, with a 2.6 m height. Grit, food and water are provided ad libitum. Three days before the experiment, birds were placed in single cages (height: 0.52 m, width: 0.46 m, depth: 0.48 m) in an indoor keeping room maintained at 22 ± 1 °C. We maintained the indoor housing room under a light regime of 14:10 h light:dark. Grit and water were provided ad libitum in the single cages whereas food was provided as stimulus during the experiment (early morning) and was subsequently provided ad libitum in each single cage until late afternoon. This ensured that animals were food motivated during the experiment in the morning. Before each trial, doves were moved in a specific testing cage (450 × 840 × 380 mm; Figure 2A) placed in the same indoor room but visually separated from the individual cages by white sheets. The bottom was covered with an anti-slip wooden panel and a branch was used as starting point in one of the short sides of the cage. The food choice area was placed in the other short side of the cage.

The guppies belong to the ‘snakeskin cobra green’ ornamental strain and were purchased from a local pet shop one month before the experiment. The fish were kept in an 80-L tank with a gravel bottom and natural plants to recreate a natural environment. The tank was illuminated by a 30-W phytostimulant lamp and set on a 12:12 h light:dark cycle. The feeding regimen consisted of two daily feedings: commercial flake food (Aquatropical, Padovan©) in the morning and live *Artemia salina* nauplii in the afternoon. Biomechanical filters and a heating system set at 26 ± 1 °C maintained a constant water condition. During the experiment, guppies were kept and tested in single tanks (250 × 400 × 250 mm; Figure 2B) for all the duration of the experiments. The tank contained 200 mm of water and had a gravel bottom as the housing tank. Two transparent plastic panels formed a pair of trapezoidal lateral compartments (80 × 50 × 200 mm each) in the middle of the tank. Each lateral compartment housed plants and one 280 × 50 mm mirror to minimize social deprivation. A 30-W phytostimulant provided illumination (12:12-h light:dark cycle). The food choice area was in the short sides of the tank opposite to the position of the fish. Flake food was provided in the morning during the experiment as stimulus whereas live food was provided in the afternoon after the experiment.

#### Stimuli

The stimuli consisted of two white plastic cards, differing in size for each species (140 × 100 mm for doves and 30 × 30 mm for guppies), presented simultaneously to the subjects. Each card displayed six black inducer circles, which were either small (8 mm diameter for doves, 2.4 mm for guppies) or large (30 mm for doves, 9 mm for guppies). In the center of each circle array, a food stimulus was placed to attract the subjects: red millet seeds for doves and commercial flake food (*Aquatropical*, Padovan©) for guppies (Supplementary Figure S1). The millet seeds were held within a white 3D-printed ring (either 12.3 mm or 15 mm in diameter, corresponding to areas of approximately 119 mm^2^ and 177 mm^2^, respectively), which was glued to the cards. For guppies, the flake food was cut into circular pieces using a scalpel to achieve consistent diameters of 3.5 mm or 4.28 mm, corresponding to areas of 9.6 mm^2^ and 14.4 mm^2^, respectively. Thus, for both species, the ratio between the areas of the two food items was 1:0.67, a common ratio used in similar previous studies (e.g., Lõoke et al., 2020; Santacà et al., 2019, 2020).

For doves, each card was presented on a L-shaped pigeon perch stand (170 × 100 mm), to ensure optimal visibility of the stimulus array from the subject’s viewpoint. For guppies, each stimulus array (composed of the card and central food stimulus) was presented by means of a transparent panel measuring 35 × 150 mm. The panel consisted of a straight vertical piece, to which a perpendicular support arm was affixed at the upper end at a 90-degree angle (Figure 2). This L-shaped design allowed the panel to rest securely on the tank edge, keeping the stimulus card in a stable and repeatable position during each trial. In a trial, two panels were positioned at opposite corners of the same short side of the tank, allowing the simultaneous view of both stimuli.

**Figure 3:**
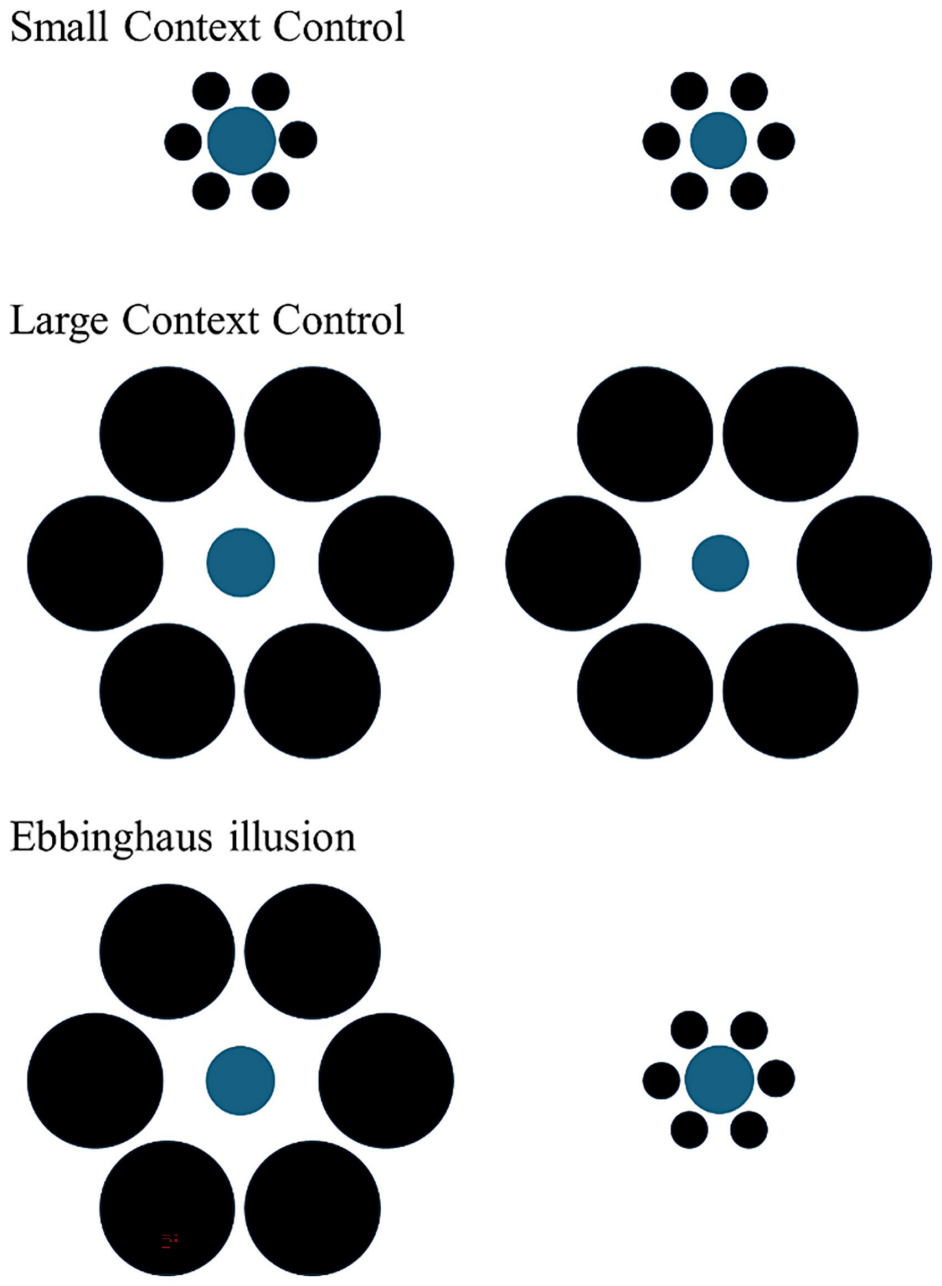
The figure shows the three different trial types used in the experiment. In both types of size discrimination trials (Small context and large control control), two different-sized food portions were presented in identical contexts. In the Ebbinghaus illusion, the same food portion appears larger to humans when surrounded by smaller inducers and smaller when surrounded by larger inducers. The size discrimination trials used in the follow-up test were identical to those in the main experiment, while the illusory trials were also the same, with the only exception being the absence of food. This design allowed us to assess whether subjects showed a spontaneous preference for inducer size when food was not present, thereby controlling for potential context-driven biases unrelated to the illusion.

In the experimental phase, subjects were presented with three different types of trial: two types of size discrimination trial (‘Small Context Control’ and ‘Large Context Control’; Figure 3) and illusory trials (Figure 3). As in previous studies (e.g., Parrish and Beran, 2014; Santacà et al., 2019, 2020), both types of size discrimination trials consisted of the presentation of two differently-sized food items, presented on two cards with same-sized inducer circles: in the Small Context Control, the inducer circles were all small whereas in the Large Context Control, all the inducer circles were large in size. Size discrimination trials were necessary to assess whether the subjects were maximizing food intake in the experimental context. In the illusory trials, we used two same-sized food items (doves: 15 mm in diameter; guppies: 4.28 mm) presented with different sized surrounding circles (Figure 2).

In this experimental design, the stimuli in the illusory trials differed in the size of the surrounding inducer circles, whereas in the size discrimination trials, the inducer circles were identical between the two stimuli. If the subjects displayed a preference for one of the two stimuli in the illusory trials, it could be suggested that they made their choice based solely on the size of the surrounding circles, ignoring the size of the food item. To rule out this possibility, a follow-up test was run one month after the main experiment. In this test, the subjects were presented with illusory control trials featuring the inducer circles from the illusory trials, but without any food stimulus. These illusory control trials were intermixed with size discrimination trials as in the experiment.

#### Procedure

In these experiments, we exploited the animals’ spontaneous preference for larger food items to assess their susceptibility to visual illusions (Santacà et al., 2021). Following previous studies that relied on this paradigm (e.g., Santacà et al., 2019, 2020), a two-step procedure was used. Animals were moved to the testing cage or tank three days before the experiment.

##### Familiarization phase

All subjects underwent a two-day familiarization phase prior to testing. This phase was necessary to habituate the animals to the cards, and to ensure they ate the food in the choice area. On the first day, subjects were presented with a single card containing some pieces of highly preferred food in the centre of the choice area: for ring doves, a central bowl containing red millet was placed between two plain white cards; for guppies, live brine shrimp were released by means of a Pasteur pipette in front of a centrally placed white card. Each subject received eight trials per day, divided into two sessions: one in the early morning and one in the late morning, with a two-hour break between sessions and a 30-min break between each trial. On the second day, the presentation of food was modified to more closely resemble the testing conditions, facilitating further habituation to the setup: for both species, two identical portions of food were presented on two cards, identical to those of the experimental phase but without any inducing circles, to prevent the development of bias toward these stimuli. As on the previous day, subjects received a total of eight trials, divided into two sessions.

For any subject that did not respond to the familiarization setup within the initial two days, the phase was extended for an additional two days. If subjects still failed to eat the food in at least 6 out of 8 trials, they were excluded from further testing.

##### Experimental phase

The experimental phase lasted six consecutive days, during which each subject underwent a total of 48 trials (32 size discrimination trials and 16 illusory trials), divided into 12 sessions of four trials each. Each day, subjects received two sessions, as in the familiarization phase, spaced two hours apart. Each trial involved the simultaneous presentation of two food stimuli and the subject was free to eat one of them. The stimulus that the subject did not choose was promptly removed whereas the chosen one was left in the apparatus until the subject consumed the food. Once the subject had consumed the food portion, the chosen stimulus was also taken out of the apparatus, and the following trial began after a 15-min interval. We scored the subject’s choice as the first card that it touched. If the subject did not choose within 15 min, the trial was considered null and repeated after a 15-min interval.

We determined the order of the trials according to a pseudo-random schedule. To prevent potential biases, no session could begin with an illusory trial, and no two consecutive trials involved the same specific stimulus configuration (e.g., same context type and same side of food presentation). In addition, the position of the larger food portion in size discrimination trials (and the position of the food portion on the smaller context in illusory trials) was counterbalanced across trials to avoid any side preference.

To rule out the possibility that subjects had a preference for the size of the inducer circles, a subsample of both doves (*N* = 12) and guppies (*N* = 10) were tested again one month after the experiment. This follow-up test was identical to the experiment with the only exception that illusory trials did not include any food stimulus (hereafter illusory control trials) as done in a similar investigation (Lucon-Xiccato et al., 2019).

##### Data analysis

Analyses were performed in R version 4.4.2 (The R Foundation for Statistical Computing, Vienna, Austria, http://www.r-project.org). We recorded accuracy (i.e., proportion of choices) in terms of selecting the larger food portion in size discrimination trials. In the illusory trials, we recorded the proportion of choices for the food portion surrounded by the small inducer circles (the ‘correct’ choice) as this is the one that humans perceive as larger. At the individual level, we used binomial tests to compare the choices for the larger food portion in size discrimination trials and for the food portion surrounded by the small inducer circles in illusory trials against chance level (0.5). We performed group analyses on the frequency of choices for the same food portions. Thus, in all analyses, ‘performance’ refers to the frequency of selecting the food portion considered correct or expected in each trial type. Not all data were normally distributed (Shapiro–Wilk test, *p* < 0.05); thus, we performed one-sample *t* tests or Wilcoxon-signed rank tests (chance level = 0.5). Cohen’s *d* (*lsr* package) was used as an effect size statistic. Furthermore, for each species separately, we used a generalized mixed-effects model for binomial distributions (GLMM, *lme4* R package) to compare performance between the different types of trials. The GLMM was also used to assess the effect of sex (only for doves, as their sample was balanced across sexes) and/or of the day. Thus, the GLMM was fitted with the type of trial, the sex and the day as fixed effects and individual ID as a random effect. For each GLMM, in the event of a significant main effect of the predictor, we tested pairwise comparisons with the Tukey *post hoc* test. We also performed a GLMM to compare the two species: the GLMM was fitted with the species, the type of trial and the day as fixed effects and individual ID nested within the species as a random effect. Model assumptions of GLMM (normality and homogeneity of the residuals) were validated by visual inspection of the residuals plotted against the fitted values and QQ-plots of residuals (Field, 2024).

The data from the follow-up test were analyzed using the same group-level and individual-level approaches as for the experiment. Pearson correlations were used to compare performance between the experimental and follow-up tests, with two specific aims: (i) to evaluate whether subjects maintained their ability to discriminate different-sized food portions in size discrimination trials, and (ii) to investigate whether performance in the illusory trials could have been influenced by spontaneous preferences for the size of the inducer circles.

## Results

**Figure 4:**
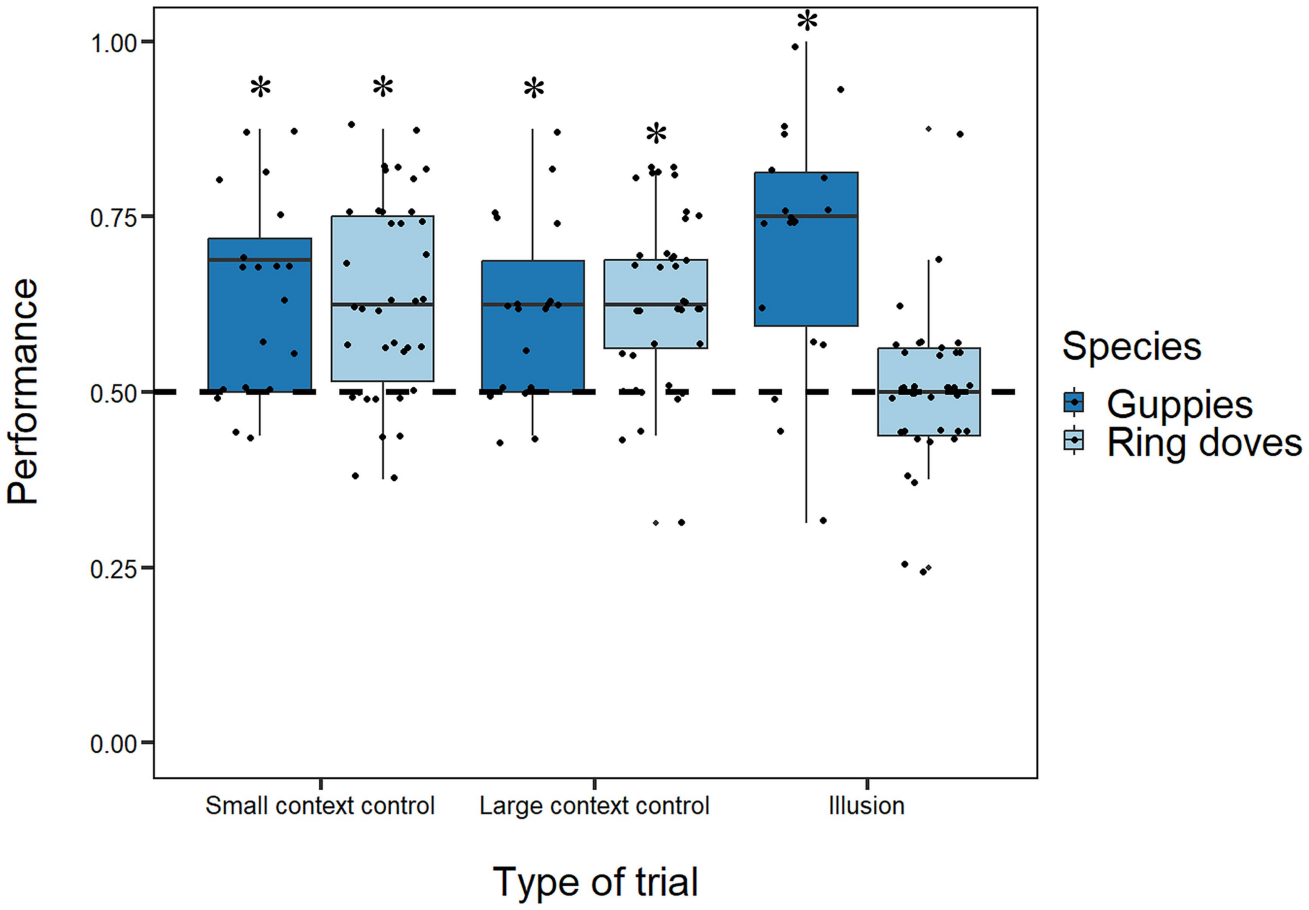
Boxplots represent median, first quartile, third quartile, ranges and outliers. The *x*-axis refers to the different types of trials (size discrimination trials and illusory trials). The *y*-axis refers to the proportion of choices for the bigger food portion in size discrimination trials and the food portion in the small context in illusory trials that is perceived as larger by humans. Asterisks (*) denote a significant departure from chance level (*p* < 0.05). Dots represent individual subjects’ performances, allowing visualization of inter-individual variability.

Binomial tests on the frequency of choices for the bigger food portion for each individual ring dove showed that of the 38 experimental subjects, 6 (3 males and 3 females) in the Large Context Control and 4 (1 male and 3 females) in the Small Context Control significantly selected the bigger food portion (Supplementary Table S1). The same analyses in guppies revealed that of 19 female subjects, 2 in the Large Context Control and 3 (1 male and 2 females) in the Small Context Control significantly selected the bigger food portion (Supplementary Table S1). Group analyses showed a significant preference of ring doves for the bigger food portion in both types of size discrimination trials [Large Context Control mean: 0.634, 95% CI (0.593, 0.674), *t*(37) = 6.663, *p* < 0.001, *d* = 1.081; Small Context Control mean: 0.637, 95% CI (0.591, 0.683), *t*(37) = 6.018, *p* < 0.001, *d* = 0.976; Figure 4]. Guppies also showed a significant preference for the bigger food portion in both types of size discrimination trials [Large Context Control mean: 0.619, 95% CI (0.558, 0.679), *t*(18) = 4.141, *p* < 0.001, *d* = 0.950; Small Context Control mean: 0.642, 95% CI (0.573, 0.710), *t*(18) = 4.333, *p* < 0.001, *d* = 0.994; Figure 4]. Paired *t*-tests revealed no difference between Large Context Control and Small Context Control in either species [ring doves: *t*(73) = 0.106, *p* = 0.916; guppies: *t*(35) = 0.534, *p* = 0.597].

In illusory trials, individual analyses showed that only one female dove and 5 guppies (1 male and 4 females) significantly chose the food portion in the small context more than chance (Supplementary Table S1). Group analyses showed a significant preference for this food portion in guppies and a lack of preference for any array in ring doves [guppies: mean = 0.714, 95% CI (0.630, 0.798), *t*(18) = 5.342, *p* < 0.001, *d* = 1.226; ring doves: mean = 0.500, 95% CI (0.465, 0.535), *t*(37) = 0.014, *p =* 0.989, *d* = 0.002; Figure 4].

Considering the ring doves, the GLMM showed that their overall performance was stable across the experimental days (*χ*^2^_1_ = 0.556, *p* = 0.465) indicating a lack of learning effect during the experiment but significantly varied as a function of the type of trials (*χ*^2^_2_ = 20.517, *p* < 0.001). A Tukey *post hoc* test showed that accuracy in size discrimination trials were significantly higher than the one in illusory trials (both *p*-values < 0.01). No difference emerged between the sexes (*χ*^2^_1_ = 1.496, *p* = 0.221) and no interaction was significant (all *p*-values > 0.648).

Considering the guppies, the GLMM showed that their overall performance was stable across the experimental days (*χ*^2^_1_ = 1.079, *p* = 0.299) and did not vary as a function of the type of trials (*χ*^2^_2_ = 5.664, *p* = 0.059) with no significant interaction (*χ*^2^_2_ = 0.700, *p* = 0.705) indicating a lack of learning effect during the experiment.

Comparing the two species, the GLMM revealed that guppies had a significantly higher overall performance than ring doves (*χ*^2^_1_ = 7.725, *p* < 0.01). The model revealed also a significant effect of the type of trials (*χ*^2^_2_ = 6.584, *p* < 0.05) with a significantly higher accuracy in size discrimination trials compared to illusory trials. Also the interaction between species and type of trials emerged as significant (*χ*^2^_2_ = 19.639, *p* < 0.001): a Tukey post hoc test showed that the two species had significantly different performance in illusory trials (*p* < 0.001) but not in size discrimination trials (both *p*-values > 0.998). Lastly, the GLMM showed that the performance was stable across the days (day: *χ*^2^_1_ = 1.441, *p* = 0.230) with no significant interaction between the species and the days (*χ*^2^_2_ = 0.040, *p* = 0.980), suggesting that both species showed similar temporal consistency.

### Follow-up test

**Figure 5:**
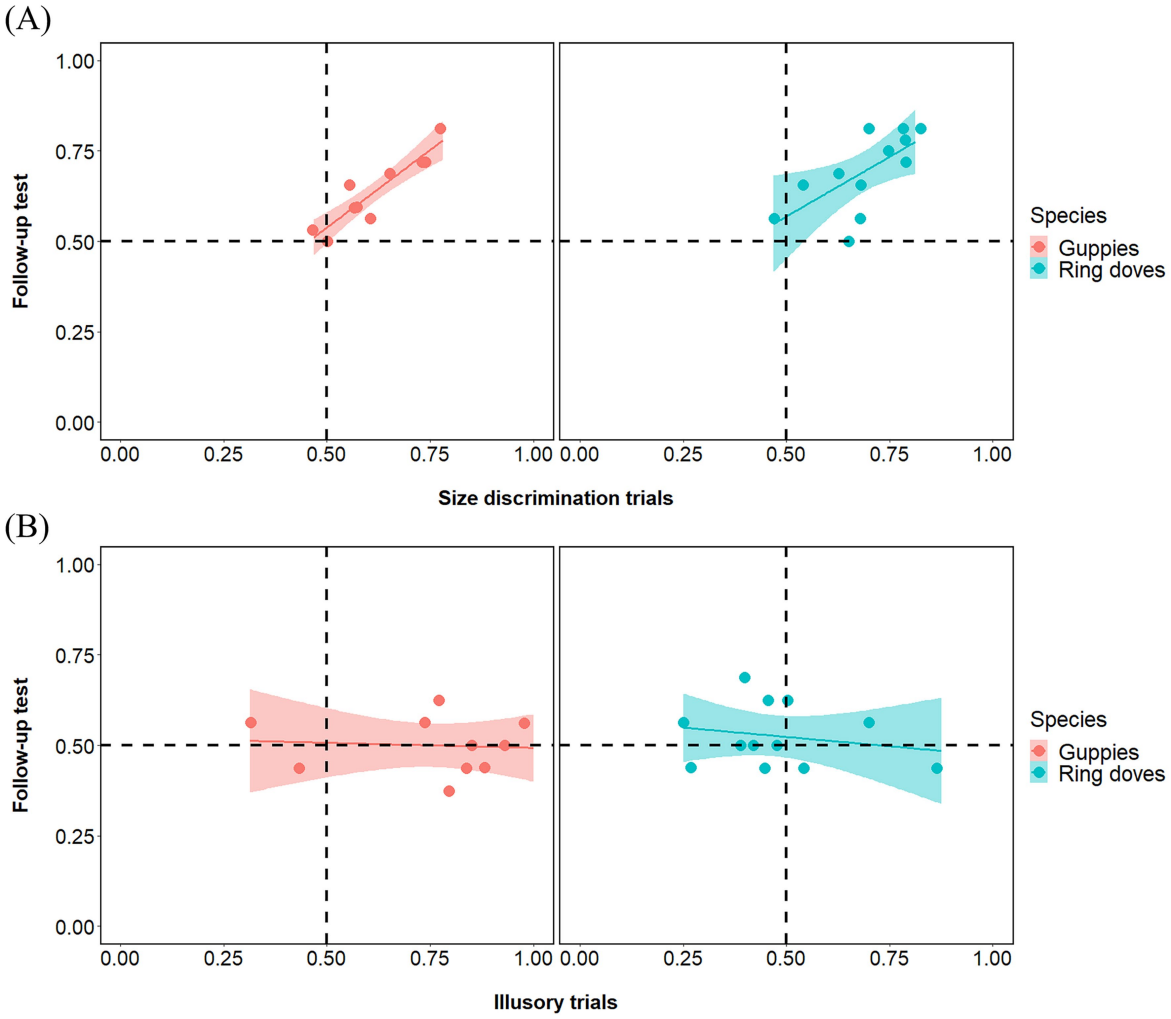
Individual-level correlations in guppies and doves between the experimental phase (x-axis) and the follow-up test (y-axis), shown separately for species and for size discrimination trials **(A)** and illusory trials **(B)**. In the experimental phase, food was present in all trials; in the follow-up test, food was removed from the illusory trials. A strong correlation in size discrimination trials confirms individual consistency in visual size preferences. In contrast, no correlation in the illusory trials suggests that choices in the experimental condition were not guided by a general preference for inducer size, supporting the conclusion that contextual elements did not drive decisions in illusory trials.

Group analyses showed a significant preference of ring doves for the bigger food portion in both types of size discrimination trials [Large Context Control mean: 0.714, 95% CI (0.628, 0.799), *t*(11) = 5.511, *p* < 0.001, *d* = 1.0591; Small Context Control mean: 0.672, 95% CI (0.594, 0.750), *Z =* 0.840, *p* < 0.01, *d* = 1.405]. Also guppies showed a significant preference for the bigger food portion in both types of size discrimination trials [Large Context Control mean: 0.638, 95% CI (0.551, 0.724), *t*(9) = 3.610, *p* < 0.01, *d* = 1.414; Small Context Control mean: 0.638, 95% CI (0.549, 0.727), *t*(9) = 3.505, *p* < 0.01, *d* = 1.108]. Paired tests revealed no difference between Large Context Control and Small Context Control in either species [ring doves: *Z =* 0.255, *p* = 0.378; guppies: *t*(18) = 0.002, *p* = 0.999]. Pearson correlations revealed that performance in size discrimination trials significantly correlated between this follow-up test and the experiment in both species (ring doves: *r*_10_ = 0.644, *p* = 0.024; guppies: *r*_8_ = 0.930, *p* < 0.001; Figure 5).

In illusory control trials (illusory trials with no food portion), group analyses did not reveal any significant preference for any array in either species [ring doves: mean: 0.526, 95% CI (0.472, 0.581), *t*(11) = 1.058, *p =* 0.313, *d* = 0.305; guppies: mean: 0.500, 95% CI (0.445, 0.556), *t*(9) = 0.008, *p* = 0.998, *d* = 0.003]. Pearson correlations revealed that performance in illusory trials did not significantly correlate between this follow-up test and the experiment (ring doves: *r*_10_ = −0.210, *p* = 0.513; guppies: *r*_8_ = −0.080, *p* = 0.827; Figure 5). This excluded the possibility that, in either species, the results of the experiment were due to any sort of spontaneous bias for the size of the inducer circles themselves.

## Discussion

This study aimed to investigate how two phylogenetically distant species, guppies and ring doves, perceive the Ebbinghaus illusion, a classic example of context-dependent size perception. By comparing these two species, we sought to explore whether susceptibility to this illusion may be shaped by shared perceptual mechanisms or reflect species-specific adaptations to different ecological contexts. Furthermore, the methodology employed in this study—a spontaneous choice paradigm—minimized training artifacts and provided insights into naturalistic perceptual tendencies (Santacà et al., 2021). By presenting biologically relevant stimuli in ecologically appropriate contexts, this approach ensures that the observed behaviors are reflective of innate perceptual strategies rather than learned responses.

The findings revealed stark interspecific differences. Guppies displayed a strong susceptibility to the illusion, consistently perceiving the central food stimulus surrounded by smaller inducers as larger, as humans do. This result corroborates previous studies demonstrating that guppies are deceived by this specific visual illusion under another ecologically relevant condition, namely during obstacle negotiation (Santacà et al., 2022). Interestingly, this susceptibility to the Ebbinghaus illusion contrasts with their response to the Delboeuf illusion (a visual illusion in which the perceived size of a central circle is influenced by the size of a surrounding concentric ring), to which guppies exhibited a reversed effect compared to humans (Lucon-Xiccato et al., 2019). The Ebbinghaus and Delboeuf illusions have often been considered similar because both involve size perception being distorted by the surrounding visual context, suggesting that they share common cognitive and neural mechanisms (Girgus et al., 1972). The difference in guppies’ responses to the Ebbinghaus and Delboeuf illusions can be attributed to the different perceptual mechanisms involved in each illusion, particularly the roles of contrast and assimilation. Both illusions require a form of global processing, but they emphasize different perceptual cues. The Ebbinghaus illusion predominantly involves assimilation, where the visual system is drawn to the surrounding context, integrating the sizes of the inducers to affect the perceived size of the central target (Girgus et al., 1972; Parrish, 2022). The effect is particularly strong when the inducers are similar to the central target, creating a perceptual “blurring” of the distinction between the target and its context. Studies have suggested that the assimilation effect in the Ebbinghaus illusion is the key factor in its strength, particularly in species that rely on holistic processing of visual stimuli (Mruczek et al., 2017; Roberts et al., 2005). The Delboeuf illusion involves both contrast and assimilation effects, which act in opposite directions. Here, the central circle is surrounded by a ring, and the perception of the size of the central circle can be influenced by whether the surrounding ring is larger or smaller. The contrast effect occurs when the surrounding ring is large, making the central circle appear smaller reflecting a perceptual tendency to exaggerate differences. Conversely, assimilation occurs when the surrounding ring is small, making the central circle appear larger reflecting a perceptual merging of target and context. The final perceptual outcome depends on which of the two mechanisms dominates in a given condition. Indeed, the interaction of both contrast and assimilation likely explains the reversed effect seen in guppies, where they tend to prioritize the assimilation effect over contrast. This may be due to their heightened sensitivity to the assimilation process, which could be an adaptation to their environment, where global integration of visual information is often more relevant than fine-grained size discrimination (Lucon-Xiccato et al., 2019). This heightened reliance on assimilation can be understood as an adaptive response to the constraints of underwater vision. In clear water, objects are often distorted by light refraction and scattered shadows, further complicating size discrimination (Luria et al., 1967). Global processing strategies that emphasize the assimilation of contextual features may mitigate these distortions by focusing on relative, rather than absolute, size cues. Such adaptations are not only ecologically relevant but also likely contribute to guppies’ remarkable capacity for rapid decision-making in competitive or predatory scenarios. Additionally, the tendency toward global processing and contextual integration, the mechanisms typically linked to assimilation in the Ebbinghaus illusion, may align with guppies’ behavioral ecology. For example, in mating contexts, females assess potential mates based on relative size within a broader visual scene, a task that may benefit from perceptual grouping processes rather than from strict analytical comparison. Similarly, during foraging or shoaling, the ability to perceive grouped stimuli may reduce the cognitive load required to evaluate food availability or social interactions, allowing for faster and more effective responses.

In contrast, ring doves did not show a significant preference in illusory trials, indicating either an absence of susceptibility to the illusion or pronounced interindividual variability. This finding is intriguing given their clear size discrimination ability in size discrimination trials. Rather than suggesting a general lack of effect in birds, our result adds to a diverse set of avian responses to the Ebbinghaus illusion. Pigeons and bantam breeds of domestic chicks have shown reversed susceptibility (Nakamura et al., 2008; Nakamura et al., 2014), newly hatched domestic chicks have exhibited human-like susceptibility (Rosa Salva et al., 2013), and European starlings have shown no group-level effect (Qadri and Cook, 2019). Such variability may partly reflect adaptations to ecological demands. For example, terrestrial granivores like doves often need to evaluate small, discrete objects—such as seeds—against heterogeneous substrates, a task that might favor attention to local detail over global context. This bias could reduce susceptibility to illusions relying on perceptual grouping, such as the Ebbinghaus illusion. However, diet alone cannot account for all avian patterns: domestic chicks, although omnivorous, feed extensively on grains and also on insects, and foraging on both food types requires the ability to detect and evaluate small items against complex backgrounds. Consequently, the relationship between feeding ecology and susceptibility to the Ebbinghaus illusion is likely to be multifaceted, and domestic chicks have in fact shown human-like susceptibility under certain conditions (Rosa Salva et al., 2013). Notably, this species represents one of the few cases in which different studies using the same illusion have yielded conflicting results: while Rosa Salva et al. (2013) reported susceptibility in four-day-old chicks, Nakamura et al. (2014) found no such effect in six-month-old birds. These inconsistencies may stem from differences in age, experimental design, or testing procedures, which can influence viewing strategies (e.g., portion of the visual field used, preferred viewing distance) and attentional focus, and may also reflect underlying individual variation in perceptual strategies, even within the same species. Interestingly, a closer examination of our individual data reveals substantial variability among doves, with some individuals perceiving the illusion similarly to humans, others in a reversed direction, and some showing no susceptibility at all (Figure 4; Supplementary Table S1). To note, while only a small number of individuals reached statistical significance in the binomial tests, this pattern is commonly observed in studies using spontaneous choice paradigms with limited trial numbers (e.g., Santacà et al., 2019, 2020). With just 16 trials per condition, a subject must make at least 13 correct choices to reach *p* < 0.05, which corresponds to over 80% accuracy. This is a high threshold, especially in untrained animals making spontaneous choices. Indeed, in size discrimination trials, most individuals showed a consistent trend in the expected direction, resulting in robust and significant group-level effects with large effect sizes (Cohen’s *d* > 1). To rule out alternative explanations for the doves’ performances in illusory trials, a follow-up test was conducted in which the inducer circles were presented without the central food stimulus. This confirmed that the doves’ (and guppies’) individual choices were guided by the visual properties of the stimulus configurations rather than a direct preference for the size of the inducer circles themselves. This leaves individual differences in perception as the most plausible explanation.

In humans, the perception of the Ebbinghaus illusion is influenced by developmental, cultural, and individual factors. Children younger than seven exhibit reduced susceptibility, as their ventral visual stream, responsible for integrating contextual cues, is still maturing (Doherty et al., 2010). Additionally, adults show significant cultural variation, with individuals from urban environments being more susceptible than those from rural settings, potentially due to differing reliance on global versus local processing in everyday visual tasks (Bremner et al., 2016). Similar variability in doves could reflect developmental stages, hormonal influences, or individual differences in neural connectivity. While the current study cannot disentangle these factors, they present intriguing possibilities for future research.

The marked variability among doves contrasts with the consistency observed in guppies, suggesting a possible influence of ecological specialization on perceptual mechanisms. This pattern aligns with previous findings in other species: redtail splitfins, which inhabit visually complex aquatic environments like guppies, show human-like susceptibility to the illusion (Sovrano et al., 2015), whereas pigeons—like ring doves—display no consistent effect (Nakamura et al., 2008). Interestingly, domestic chicks, despite being granivores like doves, have shown susceptibility under certain conditions (Rosa Salva et al., 2013), suggesting that additional factors such as age or rearing environment may also modulate perceptual strategies. Comparative studies with other bird species could provide further context. For instance, granivorous birds like doves might prioritize precision and local details in their perceptual strategies, whereas omnivorous or predatory birds may rely more heavily on global processing for tasks such as detecting prey.

These findings open several promising directions for further research. Investigating a broader range of species with varying ecological niches could help determine whether susceptibility to the Ebbinghaus illusion correlates with habitat complexity or specific sensory demands. Moreover, combining behavioral studies with neuroimaging or electrophysiological techniques could elucidate the neural mechanisms underlying these perceptual differences. Finally, exploring the role of experience and plasticity in shaping susceptibility to illusions would provide insights into the interplay between innate and learned components of perception. It is also worth considering that guppies and ring doves are not only adapted to markedly different visual environments but are also phylogenetically distant, having diverged hundreds of millions of years ago. Consequently, the perceptual differences observed here could result not only from current ecological pressures but also from traits gained or lost in ancestral lineages due to unrelated selective forces. Disentangling the relative contributions of ecological adaptation versus phylogenetic history is therefore challenging in a two-species comparison. Future research comparing more closely related species that occupy different visual environments would help to better isolate the role of ecological factors while controlling for phylogenetic relatedness. In addition, future work should address potential methodological differences in stimulus composition between species. In our design, guppies were presented with a single, unified food item cut into a circular shape, whereas doves received multiple seeds encircled by a plastic ring (Supplementary Figure S1). While this choice reflected species-specific feeding ecology and ensured motivation, it also meant that the central targets differed in visual structure, which could influence how they are processed and, consequently, susceptibility to the illusion. This factor should be considered when interpreting interspecific comparisons.

In conclusion, this study contributes to a growing body of literature on comparative visual perception, emphasizing the role of ecological context in shaping susceptibility to visual illusions. By examining guppies and ring doves—two phylogenetically distant species with distinct ecological niches—it highlights the adaptive significance of context-dependent size perception and provides a foundation for exploring broader questions about the evolution of sensory systems. These findings not only advance our understanding of perceptual strategies but also underscore the intricate interplay between ecological demands and cognitive evolution across diverse habitats.

## Data Availability

The original contributions presented in the study are included in the article/Supplementary material, further inquiries can be directed to the corresponding author/s.
