# Supplementary material for: Circles of deception: the Ebbinghaus illusion from fish to birds
Source: Front Psychol. 2025 Oct 20;16:1653695. doi: 10.3389/fpsyg.2025.1653695 (PMC12580313; doi:10.3389/fpsyg.2025.1653695)
Supplement: Supplementary file 1 [file Table_1.docx]

**
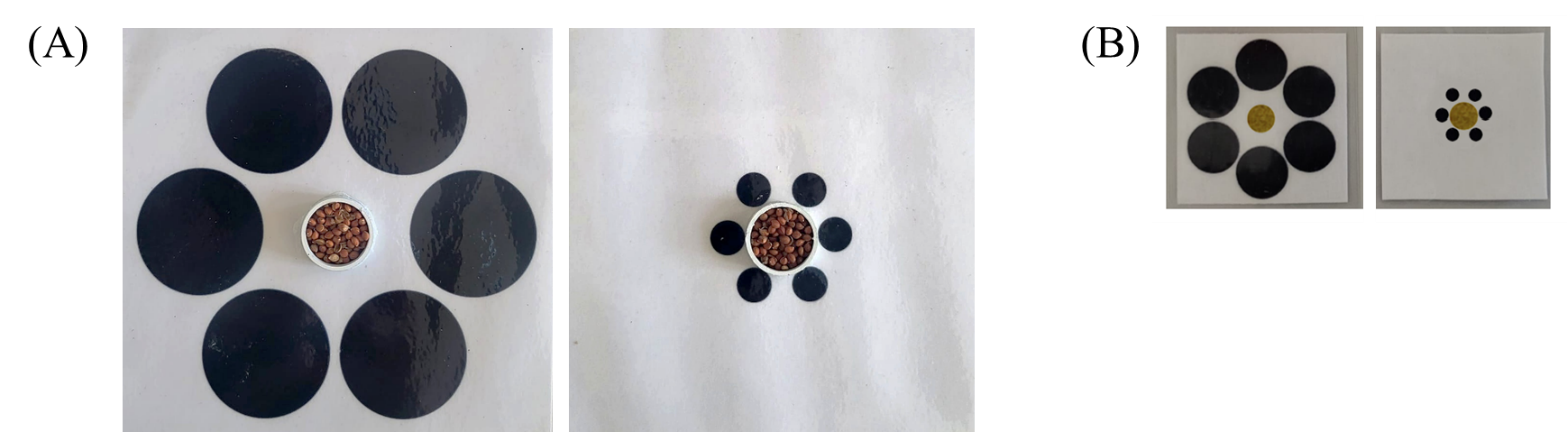
**

**Figure S1.** Photographs of the real experimental set-ups for the illusory stimuli used for the two species. In both cases, the food stimulus was placed at the centre of the Ebbinghaus configuration to allow the use of spontaneous choice tests. The left panel (A) shows the stimulus used for ring doves; the right panel (B) shows the stimulus used for guppies. Images are not to scale.

**Table S1.** Information of the subjects participating in the study and individual performance for both experimental and control phases (control trials: frequency of choices for the bigger food portion; test trials: frequency of choices for food portion surrounded by the small inducer circles for the experiment and frequency of choices for array with the small inducer circles for the control phase). Asterisks (*) denote a significant departure from chance at binomial test (*p* < 0.05).

| Subject | Species | Sex | Phase | Small Context Control | Large Context Control | Test trials |
| --- | --- | --- | --- | --- | --- | --- |
| 6 | Ring dove | F | Exp | 12/16  *p* = 0.077 | 7/16  *p* = 0.804 | 7/16  *p* = 0.804 |
| 7 | Ring dove | F | Exp | 10/16  *p* = 0.455 | 11/16  *p* = 0.210 | 8/16  *p* = 1.000 |
| 12 | Ring dove | F | Exp | 8/16  *p* = 1.000 | 8/16  *p* = 1.000 | 8/16  *p* = 1.000 |
| 16 | Ring dove | F | Exp | 9/16  *p* = 0.804 | 11/16  *p* = 0.210 | 9/16  *p* = 0.804 |
| 18 | Ring dove | F | Exp | 8/16  *p* = 1.000 | 12/16  *p* = 0.077 | 9/16  *p* = 0.804 |
| 19 | Ring dove | F | Exp | 8/16  *p* = 1.000 | 10/16  *p* = 0.455 | 8/16  *p* = 1.000 |
| 35 | Ring dove | F | Exp | 12/16  *p* = 0.077 | 11/16  *p* = 0.210 | 8/16  *p* = 1.000 |
| 40 | Ring dove | F | Exp | 14/16  *p* = 0.004* | 12/16  *p* = 0.077 | 9/16  *p* = 0.804 |
| 41 | Ring dove | F | Exp | 12/16  *p* = 0.077 | 10/16  *p* = 0.455 | 9/16  *p* = 0.804 |
| 47 | Ring dove | F | Exp | 6/16  *p* = 0.455 | 10/16  *p* = 0.455 | 9/16  *p* = 0.804 |
| 48 | Ring dove | F | Exp | 9/16  *p* = 0.804 | 10/16  *p* = 0.455 | 9/16  *p* = 0.804 |
| 49 | Ring dove | F | Exp | 13/16  *p* = 0.021* | 11/16  *p* = 0.210 | 8/16  *p* = 1.000 |
| 89 | Ring dove | F | Exp | 7/16  *p* = 0.804 | 10/16  *p* = 0.455 | 6/16  *p* = 0.455 |
|  |  |  | Control | 9/16  *p* = 0.804 | 12/16  *p* = 0.077 | 8/16  *p* = 1.000 |
| 94 | Ring dove | F | Exp | 12/16  *p* = 0.077 | 13/16  *p* = 0.021* | 14/16  *p* = 0.004* |
|  |  |  | Control | 12/16  *p* = 0.077 | 14/16  *p* = 0.004* | 7/16  *p* = 0.804 |
| 95 | Ring dove | F | Exp | 10/16  *p* = 0.455 | 12/16  *p* = 0.077 | 9/16  *p* = 0.804 |
|  |  |  | Control | 9/16  *p* = 0.804 | 9/16  *p* = 0.804 | 7/16  *p* = 0.804 |
| 99 | Ring dove | F | Exp | 8/16  *p* = 1.000 | 8/16  *p* = 1.000 | 8/16  *p* = 1.000 |
| 101 | Ring dove | F | Exp | 10/16  *p* = 0.455 | 5/16  *p* = 0.210 | 10/16  *p* = 0.455 |
| 182 | Ring dove | F | Exp | 10/16  *p* = 0.455 | 11/16  *p* = 0.210 | 7/16  *p* = 0.804 |
|  |  |  | Control | 8/16  *p* = 1.000 | 8/16  *p* = 1.000 | 8/16  *p* = 1.000 |
| 559 | Ring dove | F | Exp | 11/16  *p* = 0.210 | 11/16  *p* = 0.210 | 11/16  *p* = 0.210 |
|  |  |  | Control | 14/16  *p* = 0.004* | 12/16  *p* = 0.077 | 9/16  *p* = 0.804 |
| 575 | Ring dove | F | Exp | 8/16  *p* = 1.000 | 13/16  *p* = 0.021* | 9/16  *p* = 0.804 |
| 612 | Ring dove | F | Exp | 13/16  *p* = 0.021* | 13/16  *p* = 0.021* | 4/16  *p* = 0.077 |
|  |  |  | Control | 12/16  *p* = 0.077 | 14/16  *p* = 0.004* | 9/16  *p* = 0.804 |
| 613 | Ring dove | F | Exp | 9/16  *p* = 0.804 | 9/16  *p* = 0.804 | 8/16  *p* = 1.000 |
| 630 | Ring dove | F | Exp | 14/16  *p* = 0.004* | 10/16  *p* = 0.455 | 7/16  *p* = 0.804 |
| 700 | Ring dove | F | Exp | 12/16  *p* = 0.077 | 8/16  *p* = 1.000 | 8/16  *p* = 1.000 |
| 9 | Ring dove | M | Exp | 9/16  *p* = 0.804 | 8/16  *p* = 1.000 | 8/16  *p* = 1.000 |
| 10 | Ring dove | M | Exp | 12/16  *p* = 0.077 | 13/16  *p* = 0.021* | 7/16  *p* = 0.804 |
|  |  |  | Control | 10/16  *p* = 0.455 | 13/16  *p* = 0.021* | 10/16  *p* = 0.455 |
| 14 | Ring dove | M | Exp | 10/16  *p* = 0.455 | 10/16  *p* = 0.455 | 8/16  *p* = 1.000 |
|  |  |  | Control | 9/16  *p* = 0.804 | 13/16  *p* = 0.021* | 10/16  *p* = 0.455 |
| 34 | Ring dove | M | Exp | 7/16  *p* = 0.804 | 8/16  *p* = 1.000 | 4/16  *p* = 0.077 |
|  |  |  | Control | 9/16  *p* = 0.804 | 9/16  *p* = 0.804 | 7/16  *p* = 0.804 |
| 38 | Ring dove | M | Exp | 13/16  *p* = 0.021* | 9/16  *p* = 0.804 | 6/16  *p* = 0.455 |
|  |  |  | Control | 12/16  *p* = 0.077 | 9/16  *p* = 0.804 | 11/16  *p* = 0.210 |
| 39 | Ring dove | M | Exp | 6/16  *p* = 0.455 | 8/16  *p* = 1.000 | 8/16  *p* = 1.000 |
| 61 | Ring dove | M | Exp | 9/16  *p* = 0.804 | 9/16  *p* = 0.804 | 8/16  *p* = 1.000 |
| 88 | Ring dove | M | Exp | 8/16  *p* = 1.000 | 9/16  *p* = 0.804 | 7/16  *p* = 0.804 |
| 90 | Ring dove | M | Exp | 12/16  *p* = 0.077 | 13/16  *p* = 0.021* | 7/16  *p* = 0.804 |
|  |  |  | Control | 12/16  *p* = 0.077 | 13/16  *p* = 0.021* | 7/16  *p* = 0.804 |
| 91 | Ring dove | M | Exp | 13/16  *p* = 0.021* | 11/16  *p* = 0.210 | 8/16  *p* = 1.000 |
|  |  |  | Control | 13/16  *p* = 0.021* | 11/16  *p* = 0.210 | 8/16  *p* = 1.000 |
| 92 | Ring dove | M | Exp | 9/16  *p* = 0.804 | 11/16  *p* = 0.210 | 8/16  *p* = 1.000 |
| 96 | Ring dove | M | Exp | 11/16  *p* = 0.210 | 10/16  *p* = 0.455 | 7/16  *p* = 0.804 |
| 97 | Ring dove | M | Exp | 13/16  *p* = 0.021* | 13/16  *p* = 0.021* | 9/16  *p* = 0.804 |
| 98 | Ring dove | M | Exp | 10/16  *p* = 0.455 | 7/16  *p* = 0.804 | 7/16  *p* = 0.804 |
| 1 | Guppy | F | Exp | 11/16  *p* = 0.210 | 13/16  *p* = 0.021* | 12/16  *p* = 0.077 |
| 2 | Guppy | F | Exp | 14/16  *p* = 0.004* | 12/16  *p* = 0.077 | 12/16  *p* = 0.077 |
|  |  |  | Control | 12/16  *p* = 0.077 | 11/16  *p* = 0.210 | 7/16  *p* = 0.804 |
| 3 | Guppy | F | Exp | 8/16  *p* = 1.000 | 10/16  *p* = 0.455 | 14/16  *p* = 0.004* |
|  |  |  | Control | 7/16  *p* = 0.804 | 12/16  *p* = 0.077 | 7/16  *p* = 0.804 |
| 4 | Guppy | F | Exp | 8/16  *p* = 1.000 | 8/16  *p* = 1.000 | 12/16  *p* = 0.077 |
| 5 | Guppy | F | Exp | 10/16  *p* = 0.455 | 8/16  *p* = 1.000 | 7/16  *p* = 0.804 |
|  |  |  | Control | 10/16  *p* = 0.455 | 9/16  *p* = 0.804 | 7/16  *p* = 0.804 |
| 6 | Guppy | F | Exp | 13/16  *p* = 0.021* | 10/16  *p* = 0.455 | 10/16  *p* = 0.455 |
| 7 | Guppy | M | Exp | 7/16  *p* = 0.804 | 8/16  *p* = 1.000 | 13/16  *p* = 0.021* |
|  |  |  | Control | 8/16  *p* = 1.000 | 9/16  *p* = 0.804 | 6/16  *p* = 0.455 |
| 8 | Guppy | F | Exp | 8/16  *p* = 1.000 | 10/16  *p* = 0.455 | 15/16  *p* < 0.001* |
|  |  |  | Control | 9/16  *p* = 0.804 | 12/16  *p* = 0.077 | 8/16  *p* = 1.000 |
| 9 | Guppy | F | Exp | 12/16  *p* = 0.077 | 7/16  *p* = 0.804 | 14/16  *p* = 0.004* |
|  |  |  | Control | 10/16  *p* = 0.455 | 8/16  *p* = 1.000 | 8/16  *p* = 1.000 |
| 10 | Guppy | F | Exp | 9/16  *p* = 0.804 | 12/16  *p* = 0.077 | 9/16  *p* = 0.804 |
| 11 | Guppy | F | Exp | 7/16  *p* = 0.804 | 12/16  *p* = 0.077 | 8/16  *p* = 1.000 |
| 12 | Guppy | F | Exp | 9/16  *p* = 0.804 | 9/16  *p* = 0.804 | 12/16  *p* = 0.077 |
| 13 | Guppy | F | Exp | 11/16  *p* = 0.210 | 10/16  *p* = 0.455 | 12/16  *p* = 0.077 |
| 14 | Guppy | F | Exp | 13/16  *p* = 0.021* | 10/16  *p* = 0.455 | 16/16  *p* < 0.001* |
|  |  |  | Control | 12/16  *p* = 0.077 | 11/16  *p* = 0.210 | 9/16  *p* = 0.804 |
| 15 | Guppy | F | Exp | 8/16  *p* = 1.000 | 8/16  *p* = 1.000 | 5/16  *p* = 0.210 |
|  |  |  | Control | 9/16  *p* = 0.804 | 7/16  *p* = 0.804 | 9/16  *p* = 0.804 |
| 16 | Guppy | M | Exp | 13/16  *p* = 0.021* | 10/16  *p* = 0.455 | 12/16  *p* = 0.077 |
| 18 | Guppy | F | Exp | 10/16  *p* = 0.455 | 11/16  *p* = 0.210 | 12/16  *p* = 0.077 |
|  |  |  | Control | 10/16  *p* = 0.455 | 12/16  *p* = 0.077 | 9/16  *p* = 0.804 |
| 19 | Guppy | F | Exp | 7/16  *p* = 0.804 | 11/16  *p* = 0.210 | 10/16  *p* = 0.455 |
| 21 | Guppy | F | Exp | 11/16  *p* = 0.210 | 14/16  *p* = 0.004* | 11/16  *p* = 0.210 |
|  |  |  | Control | 13/16  *p* = 0.021* | 13/16  *p* = 0.021* | 10/16  *p* = 0.455 |
